# Hospitalization for Hypoglycemia in Japanese Diabetic Patients: A Retrospective Study Using a National Inpatient Database, 2008–2012

**DOI:** 10.1097/MD.0000000000001029

**Published:** 2015-06-26

**Authors:** Akahito Sako, Hideo Yasunaga, Hiroki Matsui, Kiyohide Fushimi, Hidetaka Hamasaki, Hisayuki Katsuyama, Tetsuro Tsujimoto, Atsushi Goto, Hidekatsu Yanai

**Affiliations:** From the Department of Internal Medicine (AS, HH, HK, H Yanai), Kohnodai Hospital, National Center for Global Health and Medicine, Ichikawa, Chiba; Department of Clinical Epidemiology and Health Economics (H Yasunaga, HM), School of Public Health, Graduate School of Medicine, University of Tokyo; Department of Health Informatics and Policy (KF), Tokyo Medical and Dental University Graduate School of Medicine; Department of Diabetes, Endocrinology, and Metabolism (TT), Center Hospital, National Center for Global Health and Medicine; and Department of Public Health (AG), Tokyo Women's Medical University, Tokyo, Japan.

## Abstract

We aimed to elucidate the epidemiology, patient demographics, and clinical outcomes of hospitalization for hypoglycemia in diabetic patients using a Japanese large-scale database.

We conducted a retrospective study using a national inpatient database of acute care hospitals in Japan. Diabetic patients ages ≥15 years with hypoglycemia as a main diagnosis for hospitalization were eligible. We estimated the annual number of hospitalizations in Japan and compared the annual admission rate by age and treatment groups. We also analyzed the association between patient characteristics and in-hospital mortality.

Among 22.7 million discharge records from July 2008 and March 2013, a total of 25,071 patients were eligible. The mean age was 73.4 years, and the mean body mass index (BMI) was 22.3 kg/m^2^. The estimated annual hospitalization for hypoglycemia in Japan was ∼20,000. Annual admission rates for hypoglycemia per 1000 diabetic patients and 1000 diabetic patients receiving insulin or oral hypoglycemic agents were 2.1 and 4.1, respectively. Patients <40 years and >70 years old were at a higher risk of hospitalization. In-hospital mortality was 3.8%, and risk factors associated with poor survival were male sex, older age, lower bed capacity, community hospital, low BMI, coma at admission, and higher Charlson Comorbidity Index.

To prevent severe hypoglycemia that leads to death and complications, individualized and careful glycemic control are important, especially in very old or young patients and in those with comorbid conditions or low BMI.

## INTRODUCTION

Diabetes is a serious and increasing global health burden. The prevalence of diabetes has been increasing recently not only in Western countries but also in non-Western countries including Asia, where the population has been growing.^1^ To prevent diabetes-related complications, intensive blood glucose control had been recommended based on large-scale studies such as the United Kingdom Prospective Diabetes Study and the Diabetes Control and Complications Trial.^2,3^ However, a recent large-scale trial showed intensive glucose-lowering therapy increased death rates and hypoglycemia, and did not significantly reduce major cardiovascular events in type 2 diabetes.^4^ A systematic review suggested that severe hypoglycemic events were associated with a higher risk of subsequent cardiovascular disease.^5^ Hypoglycemia is also associated with dementia,^6^ trauma by traffic accident, and falling.^7,8^ These findings challenge the strict glycemic control strategy in the management of diabetes. Current clinical guidelines state that a less stringent Hemoglobin A1c (HbA_1c_) goal may be appropriate for patients with a history of severe hypoglycemia, advanced vascular complications, extensive comorbid conditions, and older age associated with frailty and dementia.^9,10^

Diabetes care quality metrics established more than a decade ago have primarily focused on the prevention of hyperglycemia and its complications. However, hypoglycemia has recently been suggested as an important indicator of quality of diabetes care. Its prevention depends on physicians’ skill levels, publication of guidelines and large trials, use of medications with a lower risk of hypoglycemia, and patient education.^11^ There are some nationwide studies of incidence and temporal trends of hypoglycemia in the United States and Europe.^11–14^ However, large-scale data from outside Western countries are scarce. Race and ethnicity are associated with characteristics of diabetic patients including body mass index (BMI)^15–17^ and susceptibility to hypoglycemia.^12^ To our knowledge, little is known about the clinical course and health burden of severe hypoglycemia including incidence of concurrent ischemic heart disease and injury because most of the previous studies of hypoglycemia were either epidemiological studies without detailed clinical data in regional or nationwide populations or clinical studies conducted at a single center.

We conducted a nationwide, retrospective study using an administrative and clinical inpatient database to elucidate the incidence, patient demographics, and clinical outcomes of hospitalization for hypoglycemia in acute care hospitals in Japan.

## MATERIALS AND METHODS

### Diagnosis Procedure Combination Database

The diagnosis procedure combination (DPC) database is an administrative claims and discharge database in Japan.^18,19^ All of the 82 university academic hospitals in Japan are obliged to participate in the database, whereas participation by community hospitals is optional. Data were collected for all inpatients discharged from the participating hospitals for 6 months between July and December until 2010, and every month since 2011. The number of participating hospitals increased in 2012 to 1098 with 388,000 beds, representing ∼43% of the total bed capacity of acute care hospitals in Japan. The numbers of patient records in the database were 2.82, 2.78, 3.30, 6.96, and 6.85 million in 2008, 2009, 2010, 2011, and 2012, respectively, representing ∼50% of all discharge cases from acute care hospitals in Japan. The present study was based on a secondary analysis of the administrative claims data. The requirement for informed consent was waived because of the anonymous nature of the data. Study approval was obtained from the institutional review board of the University of Tokyo.

### Patient Selection and Variables

The DPC data include a maximum of 12 diagnoses recorded in accordance with the International Classification of Diseases, 10th Revision (ICD-10) codes and text data in the Japanese language. The maximum of 12 diagnoses consist of 4 main diagnoses, 4 comorbidities at admission, and 4 complications after admission. We retrospectively extracted the patients who had a hypoglycemia-related ICD-10 code (Table 1) in the 4 main diagnoses between fiscal year 2008 and 2012. Because ICD-10 codes of diabetic coma included hypoglycemia and hyperglycemia, and an ICD-10 code of E15 included hypoglycemia in patients with and without diabetes, we confirmed the diagnosis by Japanese text, and if necessary we reviewed the 4 diagnoses of comorbidities at admission. We excluded patients <15 years old and those without any ICD-10 code of diabetes (E10–E14) in the 12 diagnoses. Therefore, only diabetic patients ages ≥15 years admitted because of hypoglycemia were eligible for this study.

**TABLE 1:**
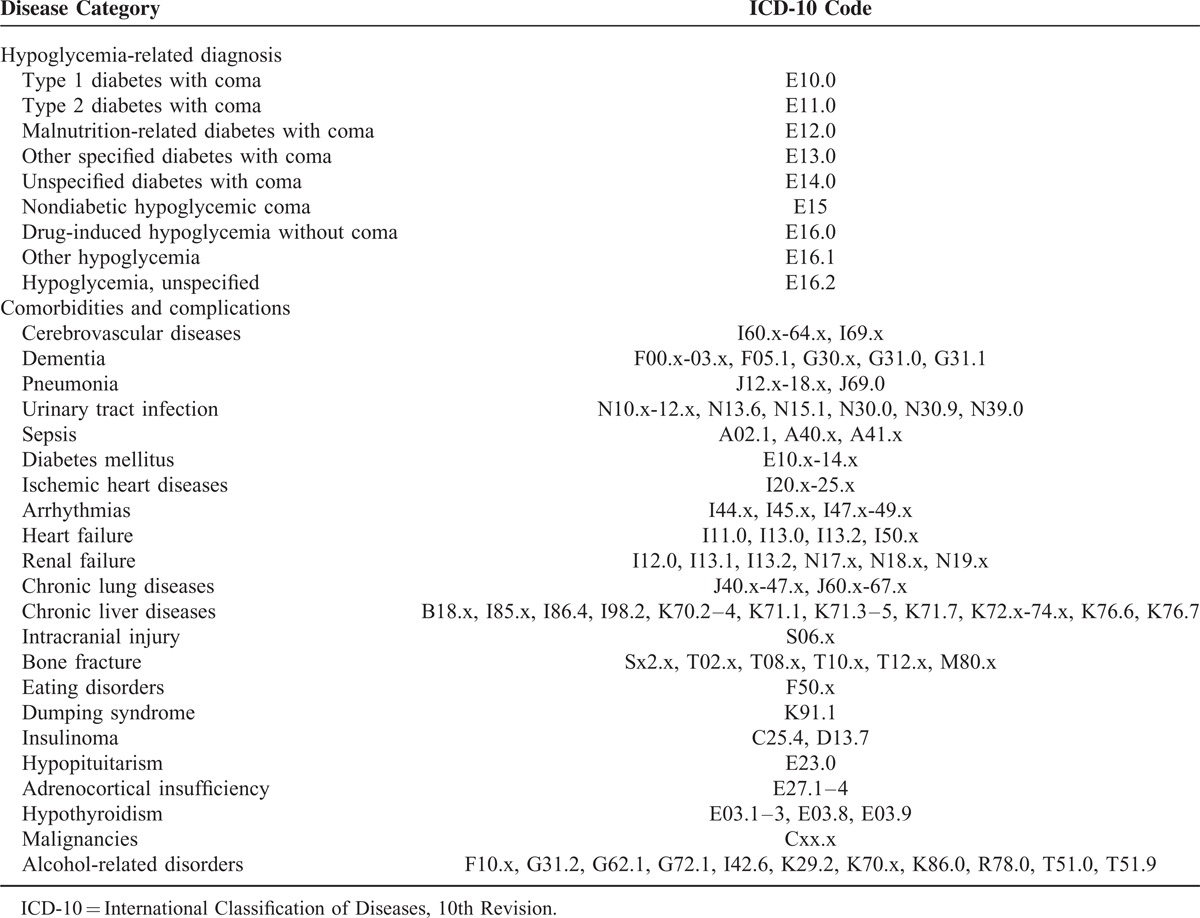
ICD-10 Codes of Underlying Diseases

We extracted the following data: age at admission, sex, height and weight (available since 2010), status at discharge, use of ambulance, unique hospital identifiers, type of hospital (academic or community), number of hospital beds, underlying disease (Table 1), procedures and surgeries recorded with the Japanese original codes, drugs prescribed during hospitalization, medical cost, and dates of admission and discharge. BMI was computed using the following standard equation: BMI = weight in kg/height squared in meters. BMI was categorized into 5 groups: underweight (BMI < 18.50 kg/m^2^), low-normal weight (18.50–22.99 kg/m^2^), high-normal weight (23.00–24.99 kg/m^2^), overweight (25.00–29.99 kg/m^2^), and obese (≥30.00 kg/m^2^).^20^ Consciousness level at admission and discharge were recorded using the Japan Coma Scale (JCS). We categorized JCS into 4 categories: grade 0 (alert); grade 1 (drowsy, but awake without any stimuli); grade 2 (somnolence, but arousable with stimulation); and grade 3 (coma).^21^ We evaluated the burden of comorbidities using the Charlson Comorbidity Index (CCI) based on the protocol of Quan.^22,23^

### Estimated Prevalence of Hospitalization for Hypoglycemia

The number of hypoglycemia hospitalizations in each fiscal year (Yi) was estimated with the following equation: 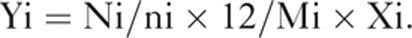


Ni is the number of beds in all acute care hospitals in Japan,^24^ ni is the number of beds in the DPC hospitals, Mi is the length of months for DPC data submission, and Xi is the observed number of hypoglycemia cases in the DPC hospitals. Hospitals were stratified by bed volume categories for the adjustment because the DPC hospitals were skewed toward larger hospitals.

The 95% confidence intervals (CIs) were calculated by Wald CIs for the population proportion: 
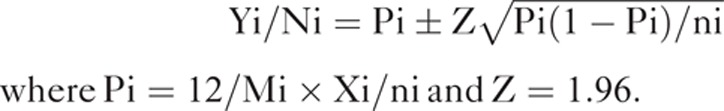


The national estimate of the population with diabetes and the population with diabetes receiving antidiabetic treatment were derived from the National Health and Nutrition Survey Japan (NHNS-J)^25^ and the Japan Population Estimates.^26^ NHNS-J is an annual cross-sectional survey of a nationally representative sample of noninstitutionalized Japanese people who are residents of 300 survey districts selected using a 2-stage stratified random sampling design.^27^ NHNS-J investigated the population with diabetes every 5 years. In NHNS-J, “individuals strongly suspected of having diabetes” were defined as those having HbA_1c_ of ≥6.5% (48 mmol/mol), or as those who responded to the questionnaire by saying that they were currently receiving diabetes treatment. “Subjects who are on medications” were defined as those who responded to the questionnaire by saying that they had used “insulin injection or any blood glucose-lowering drugs.”

### Statistical Analysis

Multivariate logistic regression analyses were performed to analyze the concurrent effects of various factors on in-hospital mortality and to determine the odds ratios and 95% CIs. We used age, sex, and variables that were clinically relevant and significantly associated with mortality in univariate analyses. All the logistic regression analyses were fitted with generalized estimating equations to take into account the dependence of the observations on the clustering effect by hospital.^28^ Values of *P* < 0.05 were considered significant. All statistical analyses were performed using IBM SPSS Statistics Version 20 (IBM SPSS, Armonk, NY).

## RESULTS

### Patient Demographic Data

From 22.7 million discharge records from the 45 months between July 2008 and March 2013, we found 81,433 patients who had any ICD-10 code of possible hypoglycemia in any of 12 diagnoses. Among them, 25,071 patients were eligible for our study criteria. The demographic data are shown in Table 2 . The mean age was 73.4 years (SD 13.1). The mean CCI was 2.5 (SD 1.7). The mean height, body weight, and BMI were 156.5 cm (SD 9.7), 54.9 kg (SD 12.3), and 22.3 kg/m^2^ (SD 4.3), respectively. Dumping syndrome and eating disorder were seen in 89 (0.4%) and 50 cases (0.2%). Insulinoma, hypopituitarism, adrenocortical insufficiency, and hypothyroidism were seen in 38 (0.2%), 97 (0.4%), 140 (0.6%), and 456 cases (1.8%), respectively.

**TABLE 2:**
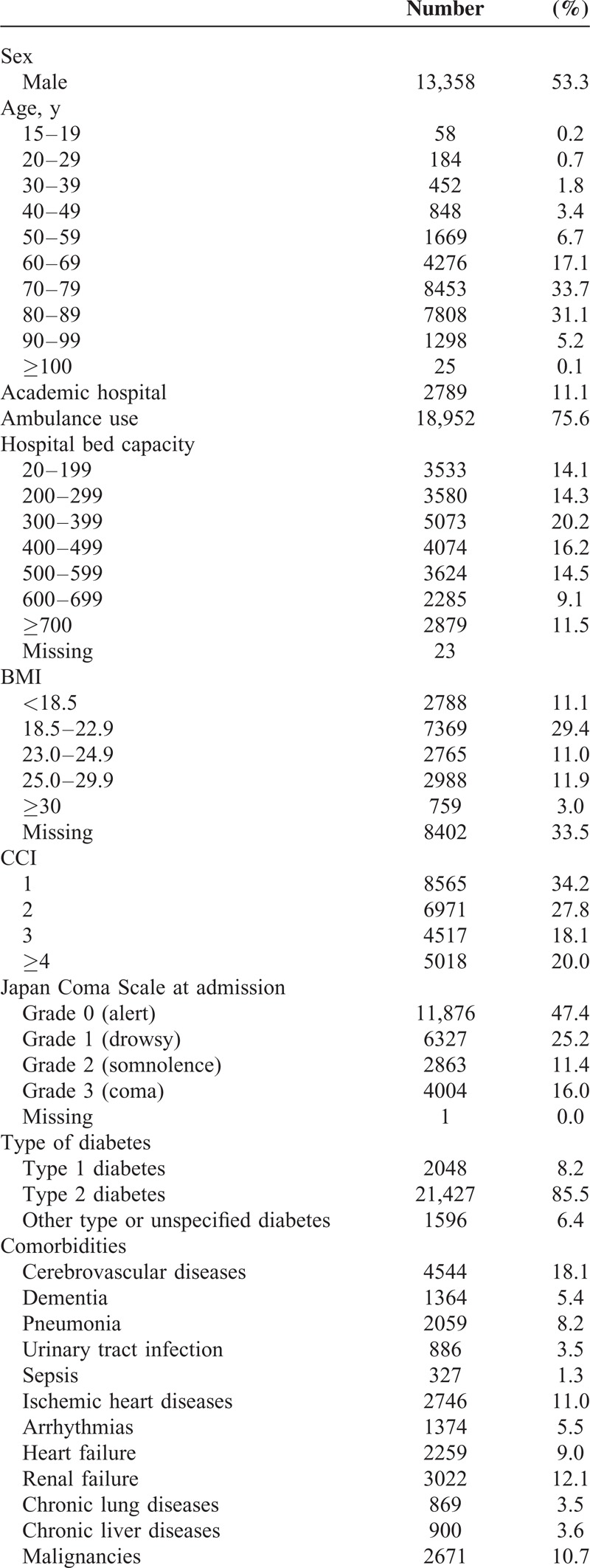
Patient Characteristics

**TABLE 2 (Continued):**
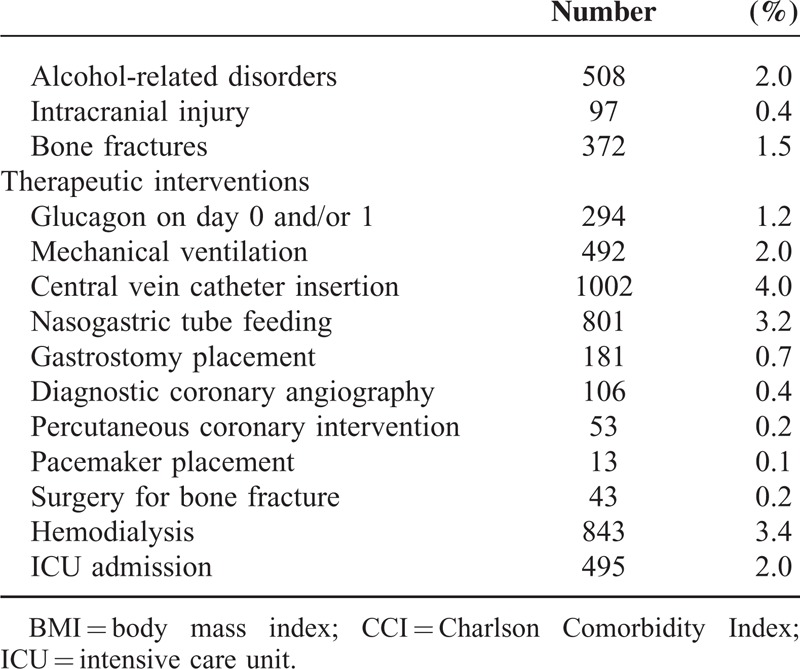
Patient Characteristics

### Prevalence of Hospitalization for Hypoglycemia

The estimated annual numbers of hospitalization for hypoglycemia in Japan were ∼20,000 (Table 3). In 2012, the proportion of hypoglycemia admissions per 1000 diabetic patients was 2.1, and the proportion of hypoglycemia admissions per 1000 diabetic patients receiving insulin and/or oral hypoglycemic agents was 4.1 (Table 4). Patients <40 years and >70 years old were at a higher risk of hospitalization.

**TABLE 3:**
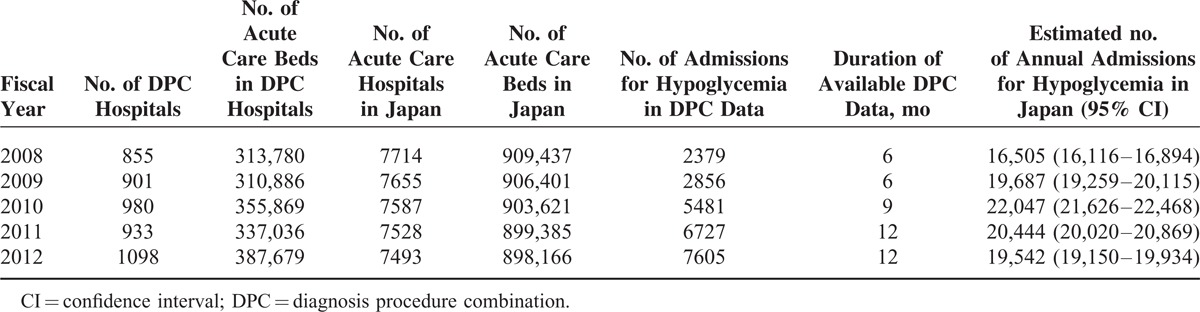
Estimated Annual Admission for Hypoglycemia In Japan

**TABLE 4:**
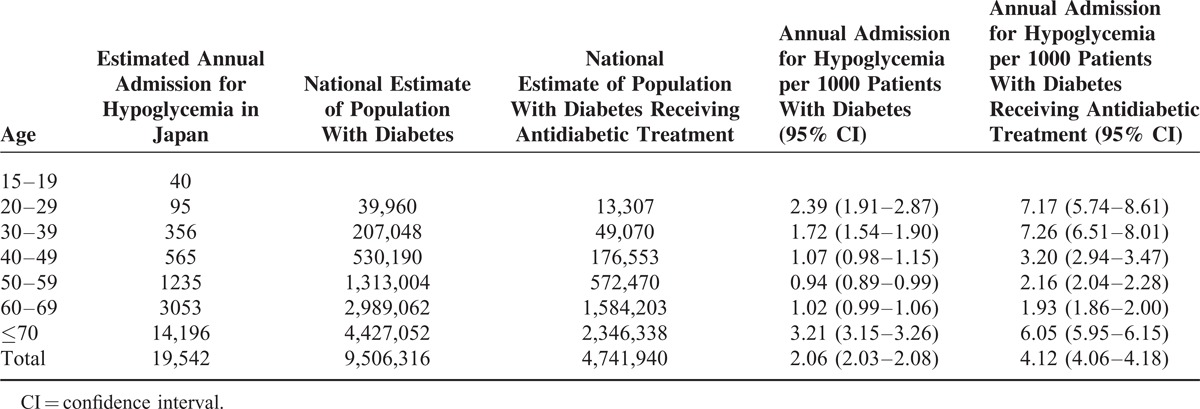
Annual Admissions for Hypoglycemia

### Clinical Outcomes and Risk Factors for In-Hospital Mortality

Median length of stay was 7 days (interquartile range 13 days). Median medical cost was 259,000 Japanese Yen (JPY) (1USD = 80 to 100 JPY during the study period; interquartile range 390,000 JPY). In terms of functional status at discharge, 1.5% had enteral feeding, 0.4% had parenteral nutrition through central venous catheter, and JCS was grade 0 for 79.8%, grade 1 for 7.9%, grade 2 for 0.8%, grade 3 for 0.5%, and missing for 11.0%.

The in-hospital mortality was 3.8%, the rate of patients discharged to home was 85.3%, and the rate of patients discharged to places other than home was 10.9%.

The results of the multivariate logistic regression analyses for in-hospital mortality are shown in Table 5. Factors associated with higher in-hospital mortality were male sex, older age, lower bed capacity hospital, nonacademic hospital, diabetes other than type 2 diabetes, underweight according to BMI, disturbance of consciousness at admission, and higher CCI.

**TABLE 5:**
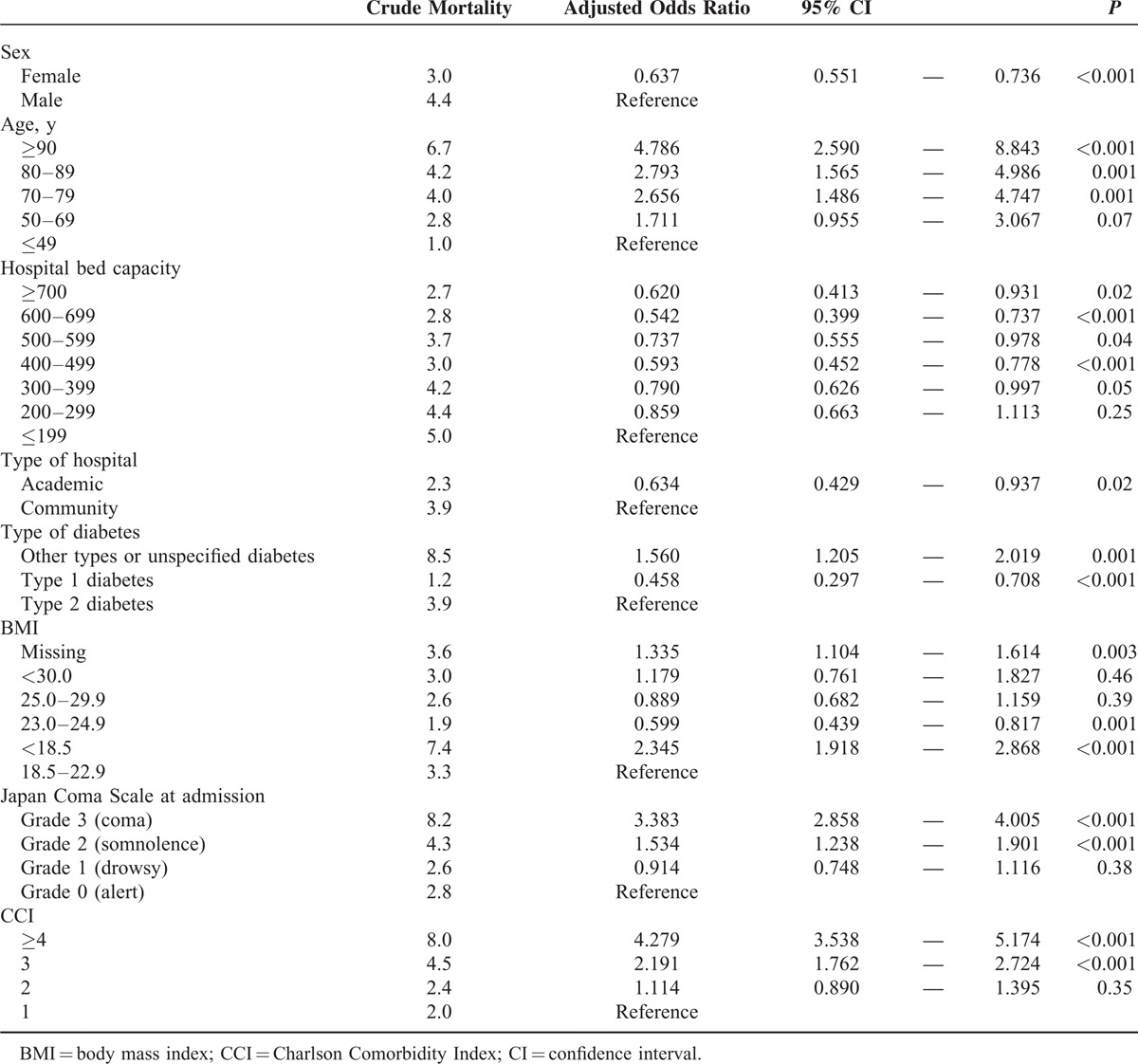
Logistic Regression Analysis for In-Hospital Mortality

## DISCUSSION

This nationwide retrospective study including 25,071 hospitalizations for hypoglycemia during 45 months investigated both epidemiological and clinical data in Japanese acute care hospitals. Mean age of the patients was 73.4 years, and ∼90% were >60 years. Mean BMI was 22.3 kg/m^2^, with 17% of patients considered underweight and 61% normal weight according to BMI. Each year, 16,000 to 22,000 diabetic patients were hospitalized for hypoglycemia, and patients <40 years and >70 years were at highest risk of hospitalization. In-hospital mortality was 3.8%, and risk factors associated with poor survival were male sex, older age, lower bed capacity, community hospital, underweight according to BMI, coma at admission, and higher CCI.

In our data, annual admission for hypoglycemia in 2012 was 2.1 per 1000 diabetic patients and 4.1 per 1000 diabetic patients receiving antidiabetic treatment. In an Italian nationwide study, the hospital admission rate for hypoglycemic coma was 0.81/1000 diabetic patients in 2001 and 0.39 in 2010.^14^ In the United States, an annual national estimate of emergency department visits per 1000 persons with diabetes receiving insulin treatment was 17.8, with one third of these requiring admission.^13^ A Canadian regional study reported that the annual rate of hospitalized hypoglycemia per 1000 people >65 years was 1.5; half of this population had diabetes and most hypoglycemia patients had diabetes.^29^ Although it is difficult to compare the incidences of hypoglycemia admission directly among different databases, health care systems, and thresholds of hospitalization, our data is the first nationwide survey in an Asian country and the largest cohort of Asian ethnicity. In the United States, a decline in hospital admissions for hypoglycemia coincided with the publication of the disappointing results of large trials about tight glycemic control.^11^ NHNS-J reported that the estimated population of “individuals strongly suspected of having diabetes” in Japan was 7.4, 8.9, and 9.5 million in 2002, 2007, and 2012, respectively. In spite of the increasing number of the diabetic patients, our data showed the decline in estimated annual admissions for hypoglycemia after 2010. This can be partly explained by the availability of antidiabetic drugs with a lower risk of hypoglycemia in Japan. At the end of 2009, dipeptidyl peptidase-4 inhibitors were approved, and metformin had been reappraised and the maximum dose increased from 750 to 2250 mg/d in 2010. Glucagon-like peptide-1 analogues were approved in 2010, and the availability of options of long-acting basal insulin and rapid-acting insulin has been increasing since 2008.

Hospital admission because of hypoglycemia in Japan may be partly attributed to inadequate home-care and prehospital care in addition to an aging population. Glucagon should be prescribed for all individuals at significant risk of severe hypoglycemia, and caregivers or family members of these individuals should be instructed on its administration.^9^ However, Japanese diabetic patients underutilize glucagon^30^ because neither physicians nor patients recognize the importance of glucagon injection and a user-friendly glucagon injection kit is not available. Most severe hypoglycemia requiring emergency ambulance services can be treated at the scene and do not need hospital care.^31^ In April 2014, the Japanese government approved emergency medical technicians with specific certification to provide blood glucose monitoring and glucose administration under a physician's supervision. This prehospital care may help diagnose and treat hypoglycemia promptly and reduce the subsequent hospitalization.

Single-center studies showed that in-hospital mortality among hypoglycemic hospitalizations was 1.4% to 7.1%.^32–35^ An Italian nationwide study based on administrative and clinical data reported that in-hospital mortality for hypoglycemic coma was 2.1%.^14^ Our data are comparable with these studies and have added several risk factors associated with mortality based on large-scale data. To our knowledge, data on the impact of BMI on the frequency and mortality of hypoglycemia are lacking. The BMI of Japanese diabetic patients reported in previous studies was considerably lower than in Western diabetic patients^16,17^ and was close to that of our study patients. We were not able to investigate the relationship between BMI and susceptibility to hypoglycemia. However, underweight according to BMI was significantly related to in-hospital death. Because advanced age, malnutrition, and poor general condition could be associated with underweight, we adjusted some of these confounders such as age and CCI. Two Japanese studies showed that diabetic patients with BMI < 18.5 kg/m^2^ had significantly higher mortality compared with normal and overweight diabetic patients.^16,36^ A cohort study of type 2 diabetes in Taiwan showed that underweight according to BMI was significantly predictive of any cause of death.^37^ Although previous studies in North America and Europe did not include patients with BMI < 18.5 kg/m^2^, they reported a U-shaped association of BMI with mortality^38^ and higher mortality in normal weight at the time of incident diabetes compared with overweight or obese.^39^ Our results suggest that hypoglycemia provides an important clue about the underlying mechanism of the association between mortality and BMI in diabetic patients.

Severe hypoglycemia is associated with a higher risk of cardiovascular diseases. Plausible mechanisms include sympathoadrenal activation, abnormal cardiac repolarization, and vasoconstriction.^5,40^ The data of the incidence of cardiovascular disease just after hypoglycemia or during hospitalization for hypoglycemia have been scarce. In our data, 0.4% of patients had diagnostic coronary angiography, 0.2% had percutaneous coronary intervention, and 0.1% had pacemaker placement during hospitalization for hypoglycemia. A single-center study in a Japanese tertiary care hospital showed that among 414 diabetic patients with severe hypoglycemia, 35% had severe hypertension, 58% had QT prolongation, 3% had newly diagnosed atrial fibrillation, 1 case had myocardial infarction requiring percutaneous coronary intervention, and 1% had stroke.^35^ Our study was comparable with these figures and provided additional data based on a large cohort. In an Italian academic hospital, 18% of type 2 diabetic patients hospitalized for severe hypoglycemia had acute coronary syndrome during hospitalization.^33^ The difference can be explained by the more frequent preexisting coronary heart disease in the Italian study and the lower incidence of ischemic heart disease in the Japanese population compared with the Western population.^15^

Hypoglycemia was also associated with a significantly higher risk of accidental falls, motor vehicle accidents,^7^ and fall-related fractures.^8^ According to the study of the National Electronic Injury Surveillance System–Cooperative Adverse Drug Event Surveillance in the United States, 5.1% of patients who visited the emergency department for insulin-related hypoglycemia and errors had suffered a fall or injury.^13^ A UK regional study showed that 2.5% of the severe hypoglycemic patients requiring emergency ambulance services had had a fall. Among 102 diabetic patients hospitalized for hypoglycemic coma in Israel, 4 had head trauma and 3 had skeletal injury.^32^ Among 414 Japanese diabetic patients with severe hypoglycemia transported to the hospital by ambulance, 5.8% had trauma, 0.5% had subarachnoid hemorrhage, and 0.5% had fracture.^35^ Although our data did not have information relating to falls and traffic accidents, this nationwide study did confirm the previous incidence data of major trauma as a serious consequence of hypoglycemia.

This study has several limitations. First, the DPC database lacked some clinical information. For instance, we had no data on laboratory tests including plasma glucose, HbA_1c_, and serum creatinine. Because we extracted the patients based on 4 main diagnoses, we may have missed hypoglycemic patients with severe comorbidities such as severe sepsis, acute coronary syndrome, renal failure, and multiple injuries even if such patients had hypoglycemia at admission. As a result, this study may have underestimated the number of annual admissions for hypoglycemia. Second, we could not assess the hypoglycemic risk of oral hypoglycemic agents and insulin because outpatient prescription data before admission were not available in the DPC database, and NHNS-J data did not specify the type of antidiabetic agents. Third, the participating hospitals in the DPC database are limited to acute care hospitals and are skewed toward large bed-volume hospitals. To adjust for this, we stratified hospitals by bed-volume categories. Although community hospitals are not obliged to participate in the DPC database, the database has ∼50% of all discharge records from acute care hospitals in Japan, and patients hospitalized in the community hospitals accounted for 88.9% of eligible patients. Fourth, the DPC database only has discharge records and does not link with other databases such as vital statistics. Therefore, we could not investigate death after discharge, and our findings are limited to short-term and in-hospital mortality. Despite these limitations, our large-scale study has elucidated the current status of hypoglycemia hospitalization in this Asian population, confirmed the results from smaller studies, and provided fundamental data to establish better clinical practice, appropriate glycemic goals, and health care policy.

In conclusion, this study showed that young and old diabetic patients had a higher risk of hospitalization for hypoglycemia compared with middle-aged patients, and burden of comorbidities, older age, and underweight according to BMI were associated with increased risk of in-hospital death. The clinical impact of complications and sequelae of hypoglycemia such as cardiovascular disease, trauma, and encephalopathy were not negligible. To prevent the severe hypoglycemia that offsets the advantages of strict glycemic control, individualized glycemic goals and careful management are important, especially in old or young patients, those with comorbid conditions, and those who are underweight according to BMI.

## References

[1] Guariguata L Whiting DR Hambleton I Global estimates of diabetes prevalence for 2013 and projections for 2035. *Diabetes Res Clin Pract* 2014; 103:137–149. 24630390 10.1016/j.diabres.2013.11.002

[2] UK Prospective Diabetes Study (UKPDS) Group. Intensive blood-glucose control with sulphonylureas or insulin compared with conventional treatment and risk of complications in patients with type 2 diabetes (UKPDS 33). *Lancet* 1998; 352:837–853. 9742976

[3] The Diabetes Control, Complications Trial Research Group. The effect of intensive treatment of diabetes on the development and progression of long-term complications in insulin-dependent diabetes mellitus. *N Engl J Med* 1993; 329:977–986. 8366922 10.1056/NEJM199309303291401

[4] Gerstein HC Miller ME Byington RP Effects of intensive glucose lowering in type 2 diabetes. *N Engl J Med* 2008; 358:2545–2559. 18539917 10.1056/NEJMoa0802743 PMC4551392

[5] Goto A Arah OA Goto M Severe hypoglycaemia and cardiovascular disease: systematic review and meta-analysis with bias analysis. *BMJ* 2013; 347:f4533. 23900314 10.1136/bmj.f4533

[6] Yaffe K Falvey CM Hamilton N Association between hypoglycemia and dementia in a biracial cohort of older adults with diabetes mellitus. *JAMA Intern Med* 2013; 173:1300–1306. 23753199 10.1001/jamainternmed.2013.6176 PMC4041621

[7] Signorovitch JE Macaulay D Diener M Hypoglycaemia and accident risk in people with type 2 diabetes mellitus treated with non-insulin antidiabetes drugs. *Diabetes Obes Metab* 2013; 15:335–341. 23121373 10.1111/dom.12031 PMC3593162

[8] Johnston SS Conner C Aagren M Association between hypoglycaemic events and fall-related fractures in Medicare-covered patients with type 2 diabetes. *Diabetes Obes Metab* 2012; 14:634–643. 22335246 10.1111/j.1463-1326.2012.01583.x

[9] American Diabetes Association. Standards of medical care in diabetes—2014. *Diabetes Care* 2014; 37 suppl 1:S14–S80. 24357209 10.2337/dc14-S014

[10] Dunning T Sinclair A Colagiuri S New IDF Guideline for managing type 2 diabetes in older people. *Diabetes Res Clin Pract* 2014; 103:538–540. 24731476 10.1016/j.diabres.2014.03.005

[11] Lipska KJ Ross JS Wang Y National trends in US hospital admissions for hyperglycemia and hypoglycemia among Medicare beneficiaries, 1999 to 2011. *JAMA Intern Med* 2014; 174:1116–1124. 24838229 10.1001/jamainternmed.2014.1824 PMC4152370

[12] Ginde AA Espinola JA Camargo CA Jr Trends and disparities in U.S. emergency department visits for hypoglycemia, 1993∗-∗2005. *Diabetes Care* 2008; 31:511–513. 18025407 10.2337/dc07-1790

[13] Geller AI Shehab N Lovegrove MC National estimates of insulin-related hypoglycemia and errors leading to emergency department visits and hospitalizations. *JAMA Intern Med* 2014; 174:678–686. 24615164 10.1001/jamainternmed.2014.136 PMC4631022

[14] Lombardo F Maggini M Gruden G Temporal trend in hospitalisations for acute diabetic complications: a nationwide study, Italy, 2001∗-∗2010. *PLoS One* 2013; 8:e63675. 23717464 10.1371/journal.pone.0063675 PMC3662780

[15] Chan JC Malik V Jia W Diabetes in Asia: epidemiology, risk factors, and pathophysiology. *JAMA* 2009; 301:2129–2140. 19470990 10.1001/jama.2009.726

[16] Tanaka S Iimuro S Akanuma Y Body mass index and mortality among Japanese patients with type 2 diabetes: pooled analysis of the Japan diabetes complications study and the Japanese elderly diabetes intervention trial. *J Clin Endocrinol Metab* 2014; 99:E2692–2696. 25202816 10.1210/jc.2014-1855

[17] Sone H Ito H Ohashi Y Japan Diabetes Complication Study Group. Obesity and type 2 diabetes in Japanese patients. *Lancet* 2003; 361:85. 12517507 10.1016/S0140-6736(03)12151-4

[18] Isogai T Yasunaga H Matsui H Effect of weekend admission for acute myocardial infarction on in-hospital mortality: a retrospective cohort study. *Int J Cardiol* 2015; 179:315–320. 25464474 10.1016/j.ijcard.2014.11.070

[19] Sako A Yasunaga H Horiguchi H Prevalence and in-hospital mortality of gastrostomy and jejunostomy in Japan: a retrospective study with a national administrative database. *Gastrointest Endosc* 2014; 80:88–96. 24472760 10.1016/j.gie.2013.12.006

[20] World Health Organization. Global Database on Body Mass Index. http://apps.who.int/bmi/index.jsp Accessed December 10, 2014.

[21] Shigematsu K Nakano H Watanabe Y The eye response test alone is sufficient to predict stroke outcome—reintroduction of Japan Coma Scale: a cohort study. *BMJ Open* 2013; 3:e002736. 10.1136/bmjopen-2013-002736 PMC3641437 23633419

[22] Charlson ME Pompei P Ales KL A new method of classifying prognostic comorbidity in longitudinal studies: development and validation. *J Chronic Dis* 1987; 40:373–383. 3558716 10.1016/0021-9681(87)90171-8

[23] Quan H Sundararajan V Halfon P Coding algorithms for defining comorbidities in ICD-9-CM and ICD-10 administrative data. *Med Care* 2005; 43:1130–1139. 16224307 10.1097/01.mlr.0000182534.19832.83

[24] Ministry of Health, Labour and Welfare, Japan. Survey on Medical Institutions [in Japanese]. http://www.e-stat.go.jp/SG1/estat/NewList.do?tid=000001030908 Accessed February 21, 2015.

[25] Ministry of Health, Labour and Welfare, Japan. Outline for the Results of the National Health and Nutrition Survey Japan, 2012 [in Japanese]. http://www.mhlw.go.jp/bunya/kenkou/eiyou/dl/h24-houkoku-05.pdf Accessed February 21, 2015.

[26] Statistics Bureau, Japan. Population Estimates. 2012 http://www.e-stat.go.jp/SG1/estat/ListE.do?lid=000001109855 Accessed February 21, 2015.

[27] Tsuboyama-Kasaoka N Takizawa A Tsubota-Utsugi M Dietary intake of nutrients with adequate intake values in the dietary reference intakes for Japanese. *J Nutr Sci Vitaminol (Tokyo)* 2013; 59:584–595. 24477258 10.3177/jnsv.59.584

[28] Hubbard AE Ahern J Fleischer NL To GEE or not to GEE: comparing population average and mixed models for estimating the associations between neighborhood risk factors and health. *Epidemiology* 2010; 21:467–474. 20220526 10.1097/EDE.0b013e3181caeb90

[29] Majumdar SR Hemmelgarn BR Lin M Hypoglycemia associated with hospitalization and adverse events in older people: population-based cohort study. *Diabetes Care* 2013; 36:3585–3590. 24089536 10.2337/dc13-0523 PMC3816904

[30] Murata T Okazaki K Yanagisawa K Glucagon underutilized among type 1 diabetes mellitus patients in Japan. *Diabetes Technol Ther* 2013; 15:748–750. 23758283 10.1089/dia.2012.0290

[31] Khunti K Fisher H Paul S Severe hypoglycaemia requiring emergency medical assistance by ambulance services in the East Midlands: a retrospective study. *Prim Care Diabetes* 2013; 7:159–165. 23375384 10.1016/j.pcd.2013.01.001

[32] Ben-Ami H Nagachandran P Mendelson A Drug-induced hypoglycemic coma in 102 diabetic patients. *Arch Intern Med* 1999; 159:281–284. 9989540 10.1001/archinte.159.3.281

[33] Fadini GP Rigato M Tiengo A Characteristics and mortality of type 2 diabetic patients hospitalized for severe iatrogenic hypoglycemia. *Diabetes Res Clin Pract* 2009; 84:267–272. 19250694 10.1016/j.diabres.2009.01.019

[34] Guven M Bayram F Guven K Evaluation of patients admitted with hypoglycaemia to a teaching hospital in Central Anatolia. *Postgrad Med J* 2000; 76:150–152. 10684324 10.1136/pmj.76.893.150 PMC1741512

[35] Tsujimoto T Yamamoto-Honda R Kajio H Vital signs, QT prolongation, and newly diagnosed cardiovascular disease during severe hypoglycemia in type 1 and type 2 diabetic patients. *Diabetes Care* 2014; 37:217–225. 23939540 10.2337/dc13-0701

[36] Yano Y Kario K Ishikawa S Associations between diabetes, leanness, and the risk of death in the Japanese general population: the Jichi Medical School Cohort Study. *Diabetes Care* 2013; 36:1186–1192. 23250802 10.2337/dc12-1736 PMC3631853

[37] Tseng CH Obesity paradox: differential effects on cancer and noncancer mortality in patients with type 2 diabetes mellitus. *Atherosclerosis* 2013; 226:186–192. 23040832 10.1016/j.atherosclerosis.2012.09.004

[38] Logue J Walker JJ Leese G Association between BMI measured within a year after diagnosis of type 2 diabetes and mortality. *Diabetes Care* 2013; 36:887–893. 23139375 10.2337/dc12-0944 PMC3609520

[39] Carnethon MR De Chavez PJ Biggs ML Association of weight status with mortality in adults with incident diabetes. *JAMA* 2012; 308:581–590. 22871870 10.1001/jama.2012.9282 PMC3467944

[40] Zoungas S Patel A Chalmers J Severe hypoglycemia and risks of vascular events and death. *N Engl J Med* 2010; 363:1410–1418. 20925543 10.1056/NEJMoa1003795

